# A Flowable Placental Formulation Prevents Bleomycin-Induced Dermal Fibrosis in Aged Mice

**DOI:** 10.3390/ijms21124242

**Published:** 2020-06-14

**Authors:** Sandeep Dhall, Anne Lerch, Nicholas Johnson, Vimal Jacob, Brielle Jones, Min Sung Park, Malathi Sathyamoorthy

**Affiliations:** Smith & Nephew Plc., Columbia, MD 21046, USA; anne.lerch@smith-nephew.com (A.L.); nicholas.johnson@smith-nephew.com (N.J.); vimal.jacob@smith-nephew.com (V.J.); brielle.jones@smith-nephew.com (B.J.); minsung.park@smith-nephew.com (M.S.P.)

**Keywords:** amnion, chorion, umbilical tissue, inflammation, oxidative stress

## Abstract

Fibrosis, the thickening and scarring of injured connective tissue, leads to a loss of organ function. Multiple cell types, including T-cells, macrophages, fibrocytes, and fibroblasts/myofibroblasts contribute to scar formation via secretion of inflammatory factors. This event results in an increase in oxidative stress and deposition of excessive extracellular matrix (ECM), characteristic of fibrosis. Further, aging is known to predispose connective tissue to fibrosis due to reduced tissue regeneration. In this study, we investigated the anti-fibrotic activity of a flowable placental formulation (FPF) using a bleomycin-induced dermal fibrosis model in aged mice. FPF consisted of placental amnion/chorion- and umbilical tissue-derived ECM and cells. The mice were injected with either FPF or PBS, followed by multiple doses of bleomycin. Histological assessment of FPF-treated skin samples revealed reduced dermal fibrosis, inflammation, and TGF-β signaling compared to the control group. Quantitative RT-PCR and Next Generation Sequencing analysis of miRNAs further confirmed anti-fibrotic changes in the FPF-treated group at both the gene and transcriptional levels. The observed modulation in miRNAs was associated with inflammation, TGF-β signaling, fibroblast proliferation, epithelial-mesenchymal transition and ECM deposition. These results demonstrate the potential of FPF in preventing fibrosis and may be of therapeutic benefit for those at higher risk of fibrosis due to wounds, aging, exposure to radiation and genetic predisposition.

## 1. Introduction

Fibrosis is pathologically characterized by excessive buildup of extracellular matrix (ECM) resulting in the thickening of injured tissue. Fibrotic or scar tissue can be found in nearly all tissue types and, in severe cases, can lead to tissue or organ dysfunction. Fibrosis in diseases such as end-stage liver disease, kidney disease, idiopathic pulmonary fibrosis, and heart failure results in fatal outcomes. Currently, no therapies are available to successfully attenuate or delay the progression of fibrosis [1]. Therefore, intervention via pharmacological or biological means, including the use of stem cells, exosomes, or paracrine factors, could lead to effective therapies to delay fibrosis progression. 

The initiation of fibrosis can arise from a myriad of stimuli, such as autoimmune reactions, infections, radiation, or chemical injury [1]. The resulting increase in secretion of inflammatory factors would lead to heightened levels of oxidative stress and an abnormal accumulation of ECM. These atypical changes are initiated by multiple cell types, including platelets, neutrophils, mast cells and macrophages, fibrocytes, fibroblasts and myofibroblasts. Furthermore, aging, a prominent fibrosis risk factor, results in a reduction in tissue regeneration. This reduction, coupled with an imbalance in ECM deposition and resorption, leads to a higher predisposition to fibrosis in aged tissue. Therefore, a major goal in the development of successful anti-fibrotic therapies is either restoration of a reparative phenotype in injured tissue or a reduction in fibrosis progression.

Placental tissue is known to be a rich source of growth factors and cytokines, hyaluronic acid-rich matrix, and endogenous viable cells, including mesenchymal stem cells (MSCs) [2,3]. MSCs have been shown to reduce inflammation, oxidative stress, and fibrosis [4,5,6,7]. Moreover, MSCs could potentially prevent fibrosis by a paracrine mechanism via the secretion of exosomes [7,8]. Hyaluronic acid, a major ECM component of placental and umbilical tissue, has been shown to suppress transforming growth factor-β (TGF-β), a collagen-inducing pro-fibrogenic cytokine, and inhibit differentiation of fibroblasts into myofibroblasts [9]. Furthermore, the placental ECM provides a reservoir of growth factors and signaling molecules that direct tissue healing and regeneration [10]. Hence, MSCs, amniotic membrane or amniotic-derived conditioned media, and umbilical tissue have been recommended as potential anti-fibrotic therapies [8,11,12,13,14]. The beneficial effects of the aforementioned anti-fibrotic therapies have been studied and proven in several organs, including the lungs [11,15], heart [16], kidney [17], liver [18], abdominal injury [14] and skin [11,19]. Therefore, the anti-inflammatory and anti-fibrotic properties of human placenta and umbilical tissue make both well-suited as therapeutic agents to prevent inflammation and fibrosis [14,20,21,22,23].

A variety of pre-clinical models exist to study both the systemic and local effects of anti-fibrotic therapies. The *in vivo* bleomycin model of fibrosis has been well established and is used to generate lung and skin fibrosis. With repeated high doses of subcutaneous bleomycin, this model can be used to investigate injury in multiple organs [24,25]. While the bleomycin model has been used extensively to study fibrosis, the majority of studies have been performed in either young mice or in adult mice [24,25]. Bleomycin-induced fibrosis studies carried out in aged mice would serve to confirm age as a crucial factor in the occurrence of fibrosis [11].

We hypothesized that a flowable placental formulation (FPF) could attenuate inflammation and fibrosis in the in *vivo* model of dermal fibrosis. To test this hypothesis, we used an aged mouse model of the well-established bleomycin-induced skin fibrosis model. We observed a reduction in inflammation and dermal hyperplasia in the presence of FPF, with no reduction being observed in its absence. Furthermore, FPF-modulated microRNA (miR) expression suggested transcriptional level changes that correlated with reduced fibrosis. Taken together, these data demonstrate the potential of FPF as a valuable therapeutic agent to prevent fibrosis in a clinical setting.

## 2. Materials and Methods

### 2.1. Tissue Procurement and Ethics Statement

Commercially available human full-term placentas were provided by the National Disease Research Interchange (NDRI, Philadelphia, PA) and Cord Blood America, Inc. (CBA, Las Vegas, NV). Tissue procurement and ethics statements were provided by NDRI and CBA. Placentas were collected after obtaining written, informed consent from eligible donors. Placentas for this study were acquired and processed within 2 days. 

### 2.2. Tissue Processing and Cryopreservation

Human normal full-term placentas were processed as previously described [22]. Briefly, amniotic membrane (AM) was separated from the chorion and umbilical cord, cleaned, and treated with an antibiotic cocktail. Chorionic membranes (CM) were treated with dispase, to loosen the membrane from the choriodecidua and decidua, and processed [21]. The trophoblast layer was mechanically separated from the chorionic membranes. The umbilical cord (UC) was cleaned and large blood vessels were removed using blades and forceps [14]. Both UC and CM were washed with saline and mechanically cleaned to remove any residual blood from the membranes, followed by soaking in an antibiotic cocktail. FPF was prepared using AM, CM, and UC through a proprietary process. Cryopreservation of FPF was performed by freezing FPF in a dimethyl sulfoxide (DMSO) (Mylan, Inc., Canonsburg, PA, USA)-containing cryoprotectant solution at a controlled cooling rate and then stored at −80 °C prior to the experiments [21].

### 2.3. Assessment of FPF Cell Viability

Fresh and cryopreserved FPF were stained with Hoechst 33342 and SYTOX Orange (Thermo Fisher Scientific, Waltham, MA, USA). A working nuclear stain was prepared by adding 2 µL of reconstituted Hoechst 33342 solution (Excitation/Emission: 350/461) and 2 µL of SYTOX Orange (Excitation/Emission: 547/570) solution to 6 µL of Dulbecco’s phosphate-buffered saline (DPBS). Of this working nuclear concentration, 1 µL was added to 1 mL of FPF for staining. Samples were briefly vortexed and incubated in the dark for 1 h. Samples were then rinsed in DPBS to remove excess staining and cell viability was evaluated using a Lionheart FX microscope with Gen5 software (BioTek, Winooski, VT, USA). Images of 64 fields at 4x magnification, in an 8 × 8 configuration, for each sample were captured for analysis. Prior to analysis, the channels were merged to generate a composite image for each field. Background fluorescence was removed from the image using a predetermined algorithm in Gen5. A fluorescent intensity threshold (FIT) for Hoechst 33342 was set to identify positively-stained cells for a total cell count. A second FIT was also set for SYTOX Orange. The second FIT identified SYTOX Orange-positive cells only from the population of Hoechst 33342-positive cells, giving a dead cell count.

### 2.4. In Vivo Testing

#### 2.4.1. Animals

A total of 10 wild-type (C57Bl/6) male mice (Jackson Laboratories, Bar Harbor, ME, USA) were used in this study. Twenty-month-old animals were housed at the Sobran BioScience vivarium at Johns Hopkins University. Experimental protocols were approved by the Sobran’s Institutional Animal Care and Use Committee. All procedures were performed in accordance with the guidelines and regulations of The Association for Assessment and Accreditation of Laboratory Animal Care International. All mice were fed a standard chow diet. 

#### 2.4.2. Dermal fibrosis model

Hair from the upper dorsa of the mice was removed using Nair (Church & Dwight Co., Inc., Ewing, NJ, USA). A 1 cm^2^ square was drawn on the midline using a marker and 100 µL of FPF (treated group) or PBS (control group) was injected onto the middle of the square. Next, 100 µL of bleomycin (0.5 mg/mL, dissolved in PBS) was injected subcutaneously every other day for 21 days. The first four injections were administered into four different corners of the square, followed by the fifth injection given at the center of the square. The bleomycin protocol was done as previously described [25]. A total of 10 injections over a period of 21 days were administered, with each “site” representing four corners of the square and the center of the square. Each site received two injections. Collected samples were either fixed in 4% paraformaldehyde and processed for histological evaluation or flash frozen and stored at −80 °C for RNA analysis.

### 2.5. Histological and Immunohistochemical Assessment

Collected wound tissue samples were fixed in 4% paraformaldehyde. Tissue sectioning and staining were performed by Premier Laboratory (Boulder, CO, USA) using standard protocols for hematoxylin and eosin (H&E), Masson’s trichrome (MT), and Picrosirius Red (PSR) staining. All slides were imaged using Aperio ScanScope AT2 (Leica biosystems, Buffalo Grove, IL, USA). Epidermal hyperplasia, dermal fibrosis, and myofiber degeneration were subjectively measured by a blinded independent pathologist. Scores were obtained by direct assessment of glass slides for all three stains, where 0 = normal tissue, 1 = minimal, 2 = mild, 3 = moderate, 4 = marked change. Dermal thickness was measured by using the measurement tool on Aperio ScanScope AT2 (Leica biosystems, Buffalo Grove, IL, USA). Samples for immunohistochemistry (IHC) were sectioned at 5 µm and mounted onto charged slides. Slides were dried overnight, baked at 60 °C for 1 h, deparaffinized in xylene, rinsed in alcohol, rehydrated in water, and equilibrated in wash buffer (TRIS buffered saline with 0.05% Tween 20; Dako, K8007). Heat-induced epitope retrieval (HIER) was performed in a Dako PT Link using a TRIS/EDTA buffer (FLEX TRS High, pH 9; Dako, K8004). For αSMA, CD3, and MAC3 protocols, the PT Link was programmed to preheat to 70 °C, followed by a constant temperature of 70 °C for 2 h after slides were added. A 20-min 95 °C retrieval was utilized for CD163b, P4HB, S100 and pSmad2. Slides were then cooled and rinsed in wash buffer and the remaining IHC steps were carried out at room temperature on an Autostainer PlusLink stainer (Dako). Within the autostainer, slides were incubated in 3.0% hydrogen peroxide for 5 min and a serum-free protein block was applied for 5 min (Dako, #X0909). The following primary antibodies and concentrations were used: for α smooth muscle actin (αSMA), rabbit polyclonal anti-αSMA primary antibody for 30 min (0.50 µg/mL) (Abcam, #ab5694); for CD3, rabbit polyclonal anti-CD3 primary antibody for 30 min (6.67 µg/mL) (Dako, #A0452); for CD163b, rabbit polyclonal anti-CD163b primary antibody for 30 min (1.376 µg/mL) (Abcam, #ab126756); for P4HB, rabbit monoclonal anti-P4HB primary antibody for 30 min (1.48 µg/mL) (Abcam, ab137110); for pSmad2, rabbit monoclonal anti-pSmad2 primary antibody for 30 min (0.85 µg/mL) (Abcam, # ab188334); for S100A4 (FSP1), rabbit monoclonal anti-S100A4 primary antibody for 30 min (0.38 µg/mL) (Abcam, #ab197896). Slides were treated with primary antibodies + EnVision + anti-Rabbit Labelled Polymer-HRP for 30 min (Dako, #K4003) and DAB+ Chromogen Solution for 5 min (Dako, K3468). Detection of MAC3 was carried out using a rat monoclonal anti-MAC3 primary antibody for 30 min (0.20 µg/mL) (BD Biosciences, #550292) and rabbit anti-rat IgG secondary antibody for 30 min (1.25 µg/mL, Abcam, #ab102248). Wash buffer rinses were carried out between appropriate reagents. All slides were then manually rinsed in tap water and counterstained for 5 min in a modified Harris hematoxylin (Dako, #S3301). The slides were rinsed again in tap water, and wash buffer was used as the hematoxylin bluing reagent. The slides were then dehydrated in absolute alcohol solutions, cleared in xylene, and placed on cover slips. All slides were imaged using Aperio ScanScope AT2 and assessed by a blinded independent pathologist. 

### 2.6. MicroRNA Array and Quantitative RT-PCR Analysis

Flash-frozen skin tissues collected at Day 21 were sent to Qiagen Inc (Germantown, MD, USA) for Quantitative RT-PCR analysis (RT^2^ qPCR) and Next Generation Sequencing (NGS) of microRNAs (miRs). 

#### 2.6.1. Sample Preparation

RNA was isolated from flash frozen tissues using the miRNeasy mini kit (QIAGEN), according to the manufacturer’s instructions, with an elution volume of 50 μL. 

#### 2.6.2. Library Preparation and Sequencing (QIAseq miR Tissue)

The library preparation was done using the QIAseq miR Library Kit (QIAGEN, Germantown, MD). A total of 100ng total RNA was converted into miR NGS libraries. Adapters containing UMIs were ligated to the RNA. Then, RNA was converted to cDNA. The cDNA was amplified using PCR (22 cycles) and, during the PCR, indices were added. After PCR, the samples were purified. Library preparation QC was performed using Bioanalyzer 2100 (Agilent, Santa Clara, CA, USA). Based on the quality of the inserts and the concentration measurements, the libraries were pooled in equimolar ratios of 4nM. The library pool(s) were quantified using qPCR. The final pool concentration that went to the sequencer was further adjusted to 1.8 pM. This library pool(s) was used to generate the clusters on the surface of a flowcell, before sequencing on a NextSeq500 instrument (SE 1 × 76 cycles) according to the manufacturer’s instructions (Illumina Inc., San Diego, CA, USA). 

#### 2.6.3. RT^2^ qPCR

RT^2^ qPCR primer assay for mouse COL1A1 (Cat# PPM05136F-200), COL1A2 (Cat# PPM26047A-200), CTGF (Cat # PPM03798B-200), TGFB1 (Cat # PPM02991B-200), TGFB2 (Cat # PPM02992A-200), TGFB3 (Cat # PPM02993A-200), SMAD2 (Cat # PPM04430C-200), SMAD3 (Cat # PPM04461C-200), ACTA2 (Cat # PPM04483A-200), IFNG (Cat # PPM03121A-200), S100A4 (Cat # PPM03811A-200), and FLI1 (Cat # PPM35556A-200) was performed. 

### 2.7. Statistical Ana Lysis

Graphpad Instat Software (Graphpad, La Jolla, CA, USA) was used for statistical analysis and plotting graphs. Results are presented as mean ± SD. A Student’s T-test was used to determine the significance of differences between groups, whereby *p* < 0.05 was considered significant. 

## 3. Results

### 3.1. Characterization of FPF

**Figure 1:**
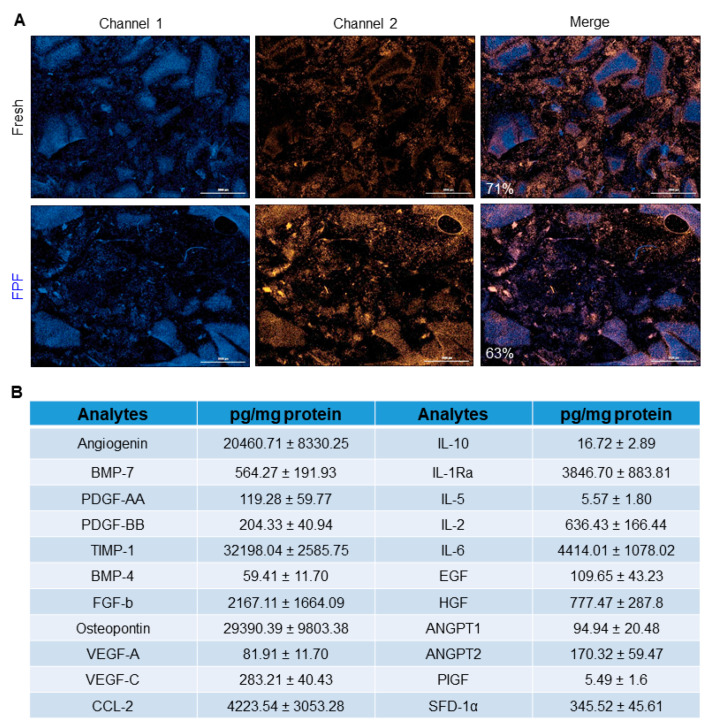
**FPF retains viable endogenous cells and beneficial factors.** (**A**) Cell viability, as determined by LIVE/DEAD staining, of fresh (*top panel*) and cryopreserved (*bottom panel*) FPF. Dead cells were visualized by SYTOX orange stain, while cell nuclei were stained with Hoechst 33342. Representative images for 1 out of 5 tested donors show a comparable number of viable cells for both conditions. Scale bars = 3000 µm **(B**) Levels of growth factors in cryopreserved FPF as determined by luminex and expressed in pg/mg protein. Detectable growth factors are as follows: angiogenin, bone morphogenetic protein (BMP-7, -4), platelet-derived growth factor (PDGF- AA, -BB), tissue inhibitor of matrix metalloproteinases-1 (TIMP-1), basic fibroblast growth factor (FGF-b), osteopontin, vascular endothelial growth factor (VEGF-A, -C), C-C motif chemokine ligand 2 (CCL-2), interleukin (IL-10, -2, -6, -5), interleukin-1 receptor antagonist (IL-1Ra), epidermal growth factor (EGF), hepatocyte growth factor (HGF), angiopoietin 1 (ANGPT1,- 2), placenta growth factor (PIGF), and stromal cell-derived factor-1α (SDF-1α). Values represent mean ± SD (N = 6).

Placenta-derived live cell delivery in *in vivo* experiments has shown to decrease the severity of fibrosis [11]. To confirm the presence of viable placental cells in FPF post-processing, we first performed the cell viability assay on fresh and cryopreserved FPF after 90 days of storage at −80 °C. Hoechst 33342 and SYTOX Orange stains confirmed the presence of viable cells in FPF post-thaw with no significant differences observed compared to freshly-prepared FPF (Figure 1A). The abundance of bioactive factors in the FPF formulation was then confirmed using multiplex analysis (Figure 1B). These factors are known for their beneficial role in wound repair and regeneration. Further, using the cryopreservation method, we have previously reported the equivalency in growth factor levels in both fresh and thawed cryopreserved tissues [14,21,26,27]. 

### 3.2. FPF Reduces Bleomycin-Induced Dermal Fibrosis in Aged Mouse Skin

**Figure 2:**
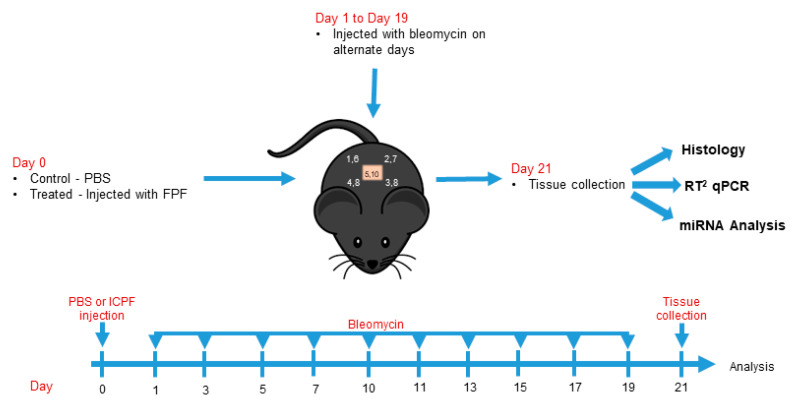
**Schematic representation of the bleomycin treatment protocol.** Animals received bleomycin injections on alternate days. Each animal received a total of 10 subdermal injections of bleomycin. Tissues collected at Day 21 were processed for histology, or flash frozen for RT^2^ qPCR and miRNA analysis. N = 5 mice/group.

Dermal fibrosis is characterized by high levels of collagen deposition and architectural changes to the epidermis and dermis. To determine whether FPF diminishes the degree of fibrosis *in vivo*, we studied bleomycin-induced dermal injury in an aged C57BL/6 mouse model. A schematic representation of the dermal fibrosis model is shown in Figure 2. On Day 0, FPF-treated mice (treated group) and PBS-treated mice (control group) were subcutaneously injected in the middle of the 1 cm^2^ marked area. Bleomycin was injected on alternate days on the marked points and tissues were collected on Day 21. 

**Figure 3:**
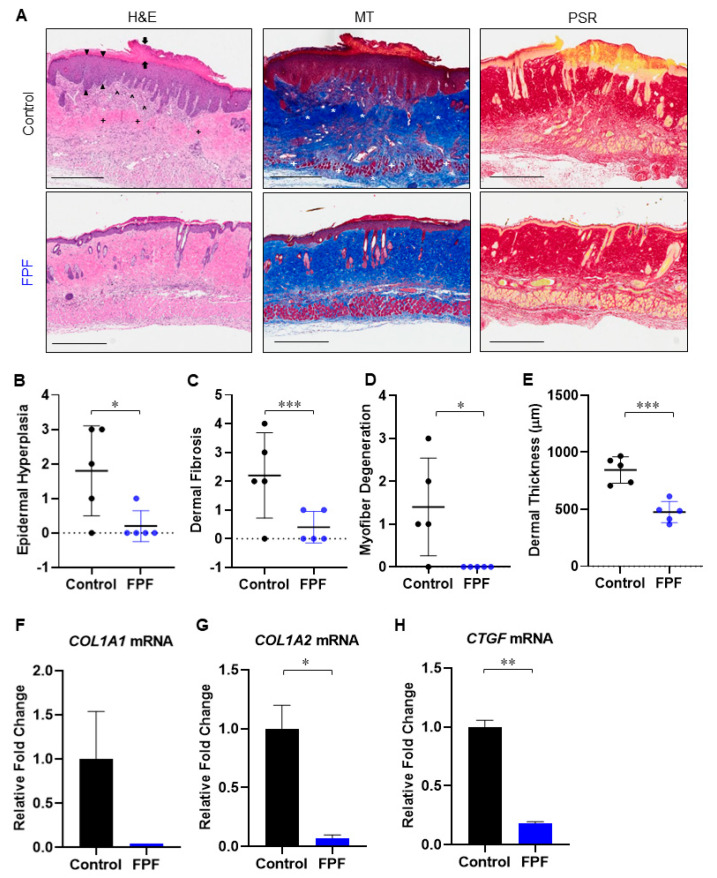
**FPF amelioratesthe progression of bleomycin-induced dermal fibrosis in aged mice.** (**A**) Histological analysis of skin tissue architecture following bleomycin injections in control (*top panels*) and FPF-treated (*bottom panels*) animals. Tissue was collected on Day 21, and tissue sections were stained with H&E (*left panels*), MT (*middle panels*), and PSR (*right panels*). Thickening in both the dermis and epidermis was observed in control mice (*top left panel, black arrows*). Conversely, fibrosis was diminished in FPF-treated skin (*bottom panel*). Arrows = Parakeratosis; Arrow heads = Epidermal hyperplasia; * = Excessive collagen deposition; + = Myofiber/degeneration/atrophy; ^ = Excessive Inflammatory cell infiltration; + = Dermal fibrosis. Representative images of five animals per group are shown. Scale bars = 500 µm. Dot plot of histological scoring for (**B**) Epidermal hyperplasia, (**C**) Dermal fibrosis, (**D**) Myofiber degeneration/atrophy, and (**E**) Dermal thickness, where 0 = normal tissue, 1 = minimal, 2 = mild, 3 = moderate, 4 = marked change. N = 5 mice/group. RT^2^ qPCR for (**F**) *COL1A1*, (**G**) *COL1A2*, (**H**) *CTGF* in control (black bars) and FPF (blue bars)-treated mice. N = 3 mice/group. * *p* < 0.05, ** *p* < 0.01, *** *p* < 0.001.

Histological examination of H&E-stained sections of the harvested skin revealed a greater magnitude of thickening in both the dermis and epidermis of control mice compared with the FPF-treated group (Figure 3A). Parakeratosis (Figure 3A, *left panel, black arrows*), epidermal hyperplasia (Figure 3A, *left panel, black arrowheads*), and dermal fibrosis (Figure 3A, *left panel, black +*), were greater in the control sections. MT-stained sections confirmed excessive collagen deposition in control skin, with more densely distributed collagen fibers in the dermis and subcutis of control mice compared with FPF-treated mice that reduced or eliminated the normal adnexal architecture in the dermis (Figure 3A, middle panel, white *). Infiltration of inflammatory cells was observed in the dermis of control skin (Figure 3A, *left panel, black ^*). Myofiber degeneration (Figure 3A, *middle panel, white +*) was also apparent in control skin. These effects were markedly reduced in the FPF-treated group. Picrosirius red (PSR) staining further confirmed the decrease in bleomycin-induced dermal fibrosis in FPF-treated mice (Figure 3A, *right panel)*. Histological scores for epidermal hyperplasia (Figure 3B), dermal fibrosis (Figure 3C) and myofiber degeneration (Figure 3D) showed significant reduction in FPF-treated mice. Similarly, dermal thickness was significantly reduced as a result of diminished fibrosis in FPF-treated mice (Figure 3E). Significant reduction in mRNA levels of collagen-encoding genes (*COL1A2* and *CTGF*), and an overall reduction in *COL1A1*, was noted using RT^2^ qPCR (Figure 3F–H). Taken together, these data demonstrate that FPF reduces the progression of bleomycin-induced fibrosis.

### 3.3. FPF Treatment Downregulates the TGF-β Pathway

**Figure 4:**
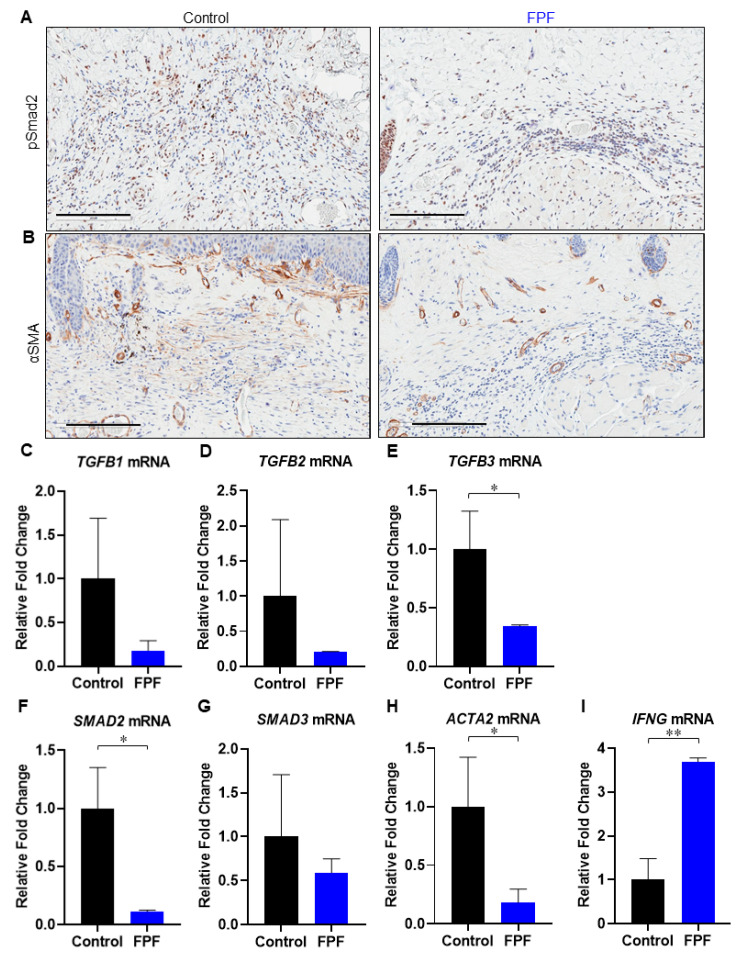
**Activation of TGF-β signaling is hindered in FPF-treated mice.** Immunohistochemical analysis of paraffin tissue sections from control (*left panel*) and FPF-treated (*right panel*) mice. (**A**) pSmad2 staining revealed fewer pSmad2-positive cells in FPF-treated mice (*right panel*) than in control mice (*left panel*), demonstrating that TGF-β signaling was disrupted following FPF treatment. (**B**) Staining for αSMA showed fewer αSMA-positive cells in FPF-treated mice (*right panel*) than in control mice (left panel), suggesting that myofibroblast differentiation was reduced following FPF treatment. Representative images of five animals per group are shown. Scale bars= 200 µm. RT^2^ qPCR for (**C**) *TGFB1*, (**D**) *TGFB2*, (**E**) *TGFB3,* (**F**) *SMAD2*, (**G**) *SMAD3*, (**H**) *ACTA2*, (**I**) *IFNG* in control (black bars) and FPF (blue bars)-treated mice. N = 3 mice/group. * *p* < 0.05, ** *p* < 0.01.

Fibrosis is primarily characterized by the overactivation of fibroblasts/ myofibroblasts and by the excessive accumulation of ECM components. Pro-inflammatory cytokine transforming growth factor-β (TGF-β) plays a critical role in regulating the pathogenesis of fibrosis. TGF-β receptor-mediated cellular responses occur via multiple intracellular components, including activated fibroblasts. TGF-β ligand–receptor complex formation results in the activation of kinases, which potentiate phosphorylation cascades involving SMAD transcription factors. Here, we examined the excised tissue by immunostaining for pSmad2 (Figure 4A). We observed an increased distribution of pSmad2-positive cells in control mice compared to FPF-treated animals (Figure 4A, *left panel*), demonstrating reduced activation of the TGF-β-SMAD2/3 pathway in FPF-treated animals.

Myofibroblast (fibroblast expressing αSMA) differentiation and tissue fibrosis have been shown to be significantly elevated in fibrotic disorders [28]. The myofibroblast phenotype is, in most cases, known to be TGF-β-dependent. We performed immunolabeling with anti-αSMA antibodies to assay for the presence of myofibroblasts in tissues undergoing fibrosis. αSMA staining revealed a marked reduction in myofibroblasts in FPF-treated mouse skin samples compared to control samples (Figure 4B, *right panel*). Skin samples were further examined using RT^2^ qPCR to see if FPF downregulated the TGF-β pathway (Figure 4 C–I). FPF-treated mice showed an overall reduced pattern in *TGFB1* (Figure 4C) and *TGFB2* (Figure 4D) and *SMAD3* (Figure 4G), and showed significant decreases in *TGFB3* (Figure 4E) and *SMAD2* (Figure 4F) expression in mRNA levels. Significant downregulation in TGF-β target gene *ACTA2* in FPF-treated mice strongly indicated a decrease in myofibroblast differentiation (Figure 4H). Furthermore, we observed a significant increase in *IFNG* (Figure 4I), which has previously been shown to inhibit collagen synthesis associated with the fibrotic response to bleomycin [29]. Taken together, these data show that FPF treatment results in downregulation of the TGF-β-mediated fibrosis pathway.

### 3.4. Infiltration of Inflammatory Cells Is Diminished in FPF-Treated Mice

**Figure 5:**
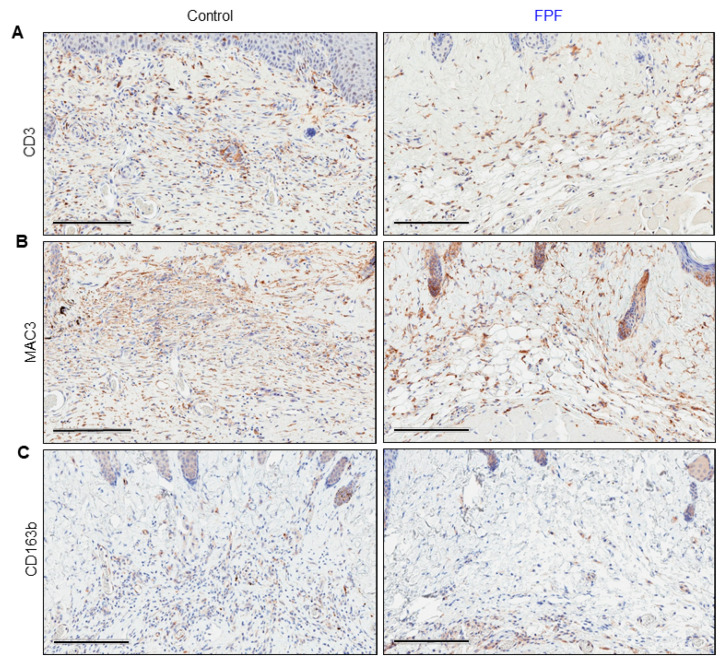
**Anti-inflammatory effect of FPF on bleomycin-treated skin.** Immunohistochemical analysis of bleomycin-treated control (*left panel*) and FPF-treated (*right panel*) tissue samples were collected at Day 21 and stained with antibodies to CD3 to detect T-cells (**A**), MAC3 to detect macrophages (**B**), and CD163b to detect M2-polarized (**C**). Amelioration of dermal tissue inflammation in FPF-treated mice compared to control mice was observed, as indicated by the lower number of CD3-positive, MAC3-positive, and CD163b-positive cells. Representative images of five animals per group are shown. Scale bars = 200 µm.

The recruitment of inflammatory cells to the site of injury works as a precursor to the cells responsible for ECM deposition and tissue remodeling. The chronic presence of inflammatory cells is hence observed in fibrotic tissues. Immunostaining of tissue sections for the presence of CD3, a T-cell marker, was done on 21-day tissue sections (Figure 5A). The degree of inflammation, as indicated by the presence of CD3-positive cells, was lower in FPF-treated mouse skin compared to control mouse skin (Figure 5A). 

Macrophages unquestionably play an important role in fibrosis. Moreover, bleomycin is known to trigger the recruitment of macrophages. We observed that mouse skin treated with FPF had less macrophage infiltration compared to the control group as assessed by MAC3, a general macrophage marker (Figure 5B). Further, we observed an increased presence of CD163b-positive cells in control mouse skin, suggesting the presence of M2 macrophages (Figure 5C). Taken together, these data show that FPF reduces the infiltration of pro-inflammatory T-cells and fibroblast-producing M2 macrophages caused by bleomycin injury.

### 3.5. FPF Decreases Fibroblast and Endothelial to Mesenchymal Transition in the Dermis 

**Figure 6:**
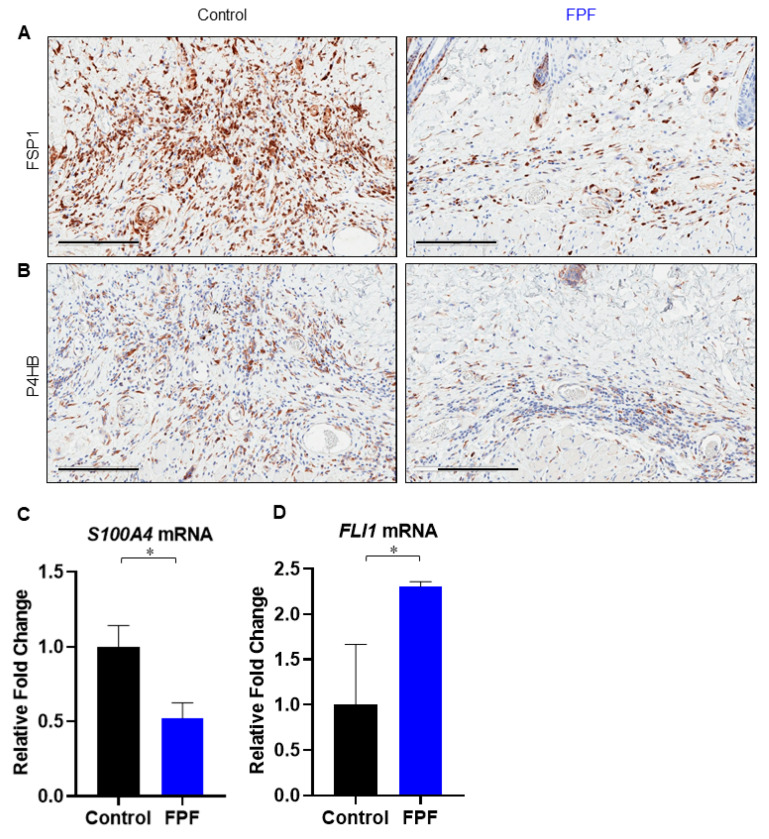
**FPF treatment reduces the number of fibroblasts and the endothelial to mesenchymal transition.** Paraffin sections from control (*left panel*) and FPF-treated (*right panel*) mice were stained with antibodies to FSP1 to detect fibroblasts (**A**) and P4HB to show cells undergoing endothelial to mesenchymal transition (**B**). The lower number of both FSP1-and P4HB-positive cells in FPF-treated mice compared to control mice was consistent with a decrease in infiltrating cells undergoing endothelial to mesenchymal transition following FPF treatment. Representative images of five animals per group are shown. Scale bars = 200 µm. RT^2^ qPCR for (**C**) *S100A4* and (**D**) *FLI1* in control (black bars) and FPF (blue bars)-treated mice. N = 3 mice/group. * *p* < 0.05.

Fibrocyte activation and endothelial to mesenchymal transition (EndMT) have been shown to occur in areas of tissue injury and inflammation. These two processes contribute to the generation of fibrotic tissue. The high concentration of fibroblasts in control mice compared to FPF-treated animals was confirmed by staining skin cryosections for Fibroblast Specific Protein 1 (FSP1), also known as S100A4 (Figure 6A). FSP-1 is associated with cells of mesenchymal origin or of fibroblastic phenotype. Moreover, mesenchymal marker prolyl 4-hydroxylase (P4HB) was markedly reduced in FPF-treated tissue compared to control tissue, consistent with the observed decrease in fibroblasts in FPF-treated mouse skin (Figure 6B). RT^2^ qPCR results for *S100A4*, a biomarker of type II epithelial to mesenchymal transition (EMT) [30], was found to be significantly reduced in FPF-treated mice (Figure 6C). *FLI1*, another predisposing factor in dermal fibrosis and systemic sclerosis, is constitutively downregulated in skin lesions of patients [31,32]. Moreover, *FLI1* haploinsufficiency induces EndMT, and its expression was significantly upregulated in the FPF-treated mice (Figure 6D) [31,33]. These data suggest that FPF treatment results in dampened infiltration of fibroblasts and that FPF could potentially result in the reduction in EMT and EndMT.

### 3.6. FPF Treatment Improves Transcriptional Dysregulation in Fibrosis Disease Pathogenesis

**Figure 7:**
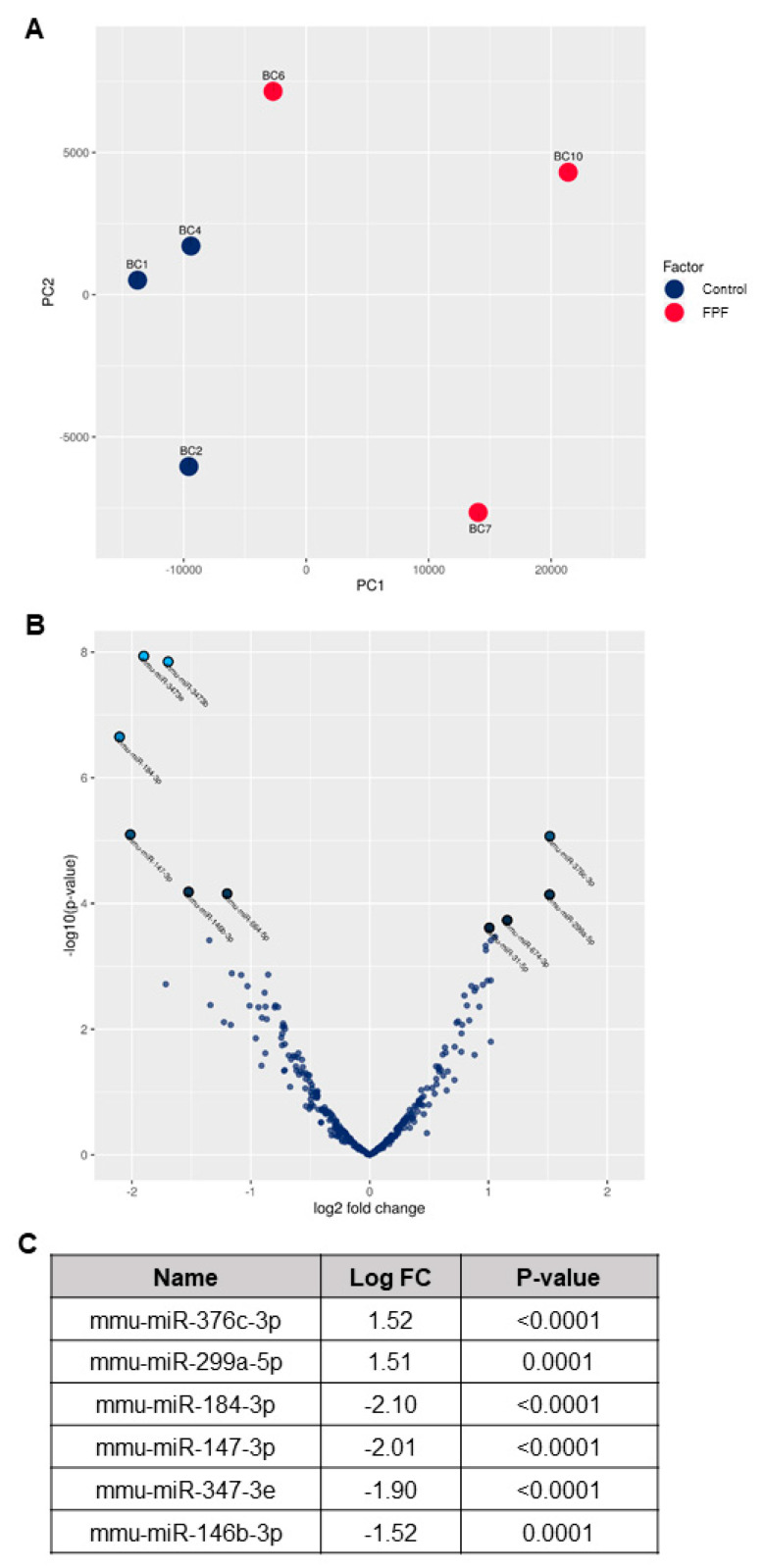
**FPF modulates transcriptional regulation of fibrosis.** Next Generation Sequencing for miRs was performed on flash-frozen tissue sections prepared from control and FPF-treated mice (n = 3/group). (**A**) Principal component analysis (PCA) showing the distance and relatedness between samples, with 50 miRs having the largest coefficient of variation. (**B**) Volcano plot showing the relationship between p-values and fold change in normalized expression between control and FPF-treated groups. (**C**) Most differentially-expressed known miRs. A complete list of the 25 most significantly differentially-expressed miRs and annotation, with log fold change (logFC) between control and FPF-treated groups is shown in Supplementary Table S1. N = 3 mice/group.

Previous literature has established that microRNA (miR) dysregulation plays an important role in fibrotic disease pathogenesis and progression [34]. To explore this possibility, we performed Next Generation Sequencing (NGS) to identify miRs and compare tissue-specific miR expression in FPF-treated and control mice. To begin these analyses, a principal component analysis (PCA) was performed to identify the two principal components (PC1 and PC2) (Figure 7A). Some separation within the treated samples was observed. However, two independent clusters for control and FPF-treated groups were readily apparent. A Volcano Plot was constructed to identify statistically significant differentially-expressed miRs (Figure 7B). The two regions of interest are the top of the plot, indicating miRs with a high statistical significance, and the far left and right of the plot, highlighting those miRs that are either strongly down- or up-regulated, respectively. The criterion used to screen down- or up-regulated miRs was a log fold change ≥1.5 and a *p* value ≤ 0.0001. Based on these analyses, the most differentially-expressed known miRs are mmu-miR-376c-3p (up-regulated), mmu-miR-299a-5p (up-regulated), mmu-miR-184-3p (down-regulated), mmu-miR-147-3p (down-regulated), mmu-miR-3473e (down-regulated), and mmu-miR-146b-3p (down-regulated) (Figure 7C, Supplementary Table S1). These differentially-expressed miRs are known for their role in the regulation of inflammation, TGF-β signaling, extracellular matrix production and deposition, fibroblast proliferation, and EndMT, and are altered in control mouse tissues. Hence, the data strongly suggests that FPF favorably modulates miRs involved in the onset and progression of fibrosis, leading to a reduction in tissue injury.

## 4. Discussion

Fibrosis, the thickening and scarring of injured connective tissue, can lead to tissue and organ dysfunction and disease. The inflammatory response is tightly linked to tissue injury that initiates a cascade of detrimental processes leading to tissue fibrosis. The classical evaluation of tissue fibrosis is done via histology of the suspected tissue. Oftentimes, certain indications, such as skin scleroderma, can be visually predicted. However, deeper assessment of improper collagen deposition and excessive inflammation is visualized histologically. In this study, we evaluated the potential of FPF, rich in all the endogenous bioactive components of the placental membranes, to reduce the progression of fibrosis in a bleomycin-induced dermal mouse model using aged mice. We observed that FPF treatment inhibited fibrosis in this model. Moreover, we observed a reduction in inflammation, TGF-β signaling, fibroblast proliferation and ECM deposition in FPF-treated animals. Additionally, we observed changes at the transcriptional level as RT^2^ qPCR and NGS analysis revealed modulation in fibrosis-specific mRNAs and miRs, respectively, in FPF-treated animals.

Inflammation and fibrosis are the top hallmarks of many disease states. In wound healing, the derailment of inflammatory processes leads to chronicity and fibrotic tissue formation. Furthermore, age critically influences abnormal wound responses that result in excessive ECM formation, leading to tissue fibrosis. Here, we used the bleomycin model of skin fibrosis in aged mice. Bleomycin, a copper-chelating anti-tumor peptide that can cleave DNA, was used to create dermal injury. In the tissue microenvironment, bleomycin leads to the release of reactive oxygen species and the recruitment of inflammatory cells, which in turn activates the resident fibroblasts and stimulates ECM formation. Therefore, a subcutaneous injection of bleomycin can trigger skin fibrosis that persists for up to six weeks [25]. The characteristics of this model have also been shown to mimic features of scleroderma [24]. Moreover, repeated doses of subcutaneous bleomycin can cause injury to multiple organs, including the skin, lung, and gastrointestinal tract [24]. Nonetheless, the majority of previous studies have been carried out with young mice, which are better able to recover from bleomycin-induced injury than their older counterparts. Although we used a previously published procedure to induce dermal fibrosis in this work, to the best of our knowledge, this study is the first to report the use of an aged murine model of dermal fibrosis, which is more representative of the aged human population susceptible to fibrotic disorders. Thus, this model can be used to investigate anti-fibrotic therapies relevant to patients with fibrosis.

Given the complexity of fibrosis and the dysregulation of multiple pathways, the development of effective anti-fibrotic therapies demands a multifactorial therapeutic approach. Placental tissue is a rich source of cytokines, growth factors and endogenous cells that provide a variety of benefits [3,13,35]. The viability of native cells in FPF post-thaw, similar to our previous studies, is retained and comparable to a fresh formulation (Figure 1A) [14,21,22]. These native cells, derived from placental tissues and umbilical cord, could be a contributing factor to alleviate fibrosis by regulating the expression of various tissue pro- and anti-fibrotic factors [5,36,37,38]. The placental cells are surrounded and embedded in ECM, which is a reservoir rich in a mixture of proteins and signaling molecules that are released by cells as they grow. Recent studies show that ECM is no longer just a structural scaffold, but is actively involved in providing signaling cues and plays a dynamic role in host cellular behavior to repair damaged tissue [10]. Further, the plethora of beneficial growth factors and cytokines present in the FPF (Figure 1B) are conducive to reducing inflammation and fibrosis [39,40,41]. Since the cocktail of signaling molecules and proteins are embedded in the ECM and cells within, FPF could impart a sustained release of these valuable factors. In addition, we have previously shown that our method of tissue preservation retains all the growth factors present in the fresh tissue [14,21,26,27]. Overall, these features make FPF a viable option for therapeutic applications.

The reduced levels of epidermal fibrosis observed in FPF-treated mice provide support for its potential to regulate keratinocyte activation and differentiation, both of which remain at the core of sclerosis, dermal fibrosis, and non-healing wounds [42]. Our data further indicate that treatment with FPF prior to bleomycin treatment inhibited parakeratosis and subcutis fibrosis (Figure 3A). Importantly, there was a marked difference in dermal fibrosis and dermal thickness, epidermal hyperplasia, collagen deposition and myofiber atrophy between FPF-treated and control animals. Consistent with this data, we also observed downregulation of *COL* genes and *CTGF*, both well-known downstream mediators of pro-fibrotic TGF-β [28,43,44,45]. These results could be due to the paracrine effects of the placental tissue, including a strong inhibition of fibroblast activation and diminished collagen synthesis [8]. 

TGF-β is a master cytokine that regulates tissue fibrosis via the activation of fibroblasts, differentiation into myofibroblasts, and stimulation of excessive ECM production. TGF-β ligand–receptor complex formation results in the activation of kinases that potentiate SMAD transcription factors and a phosphorylation cascade. Downstream, the activation of SMAD up-regulates the expression of several profibrotic genes, including collagens, as highlighted in the thickened dermis of control mice (Figure 3A,E) [46]. Disproportionate levels of collagen deposition is a prominent feature observed in impaired excisional or burn wounds, scarring, and various fibrotic disorders. Myofibroblasts differentiation and accumulation contribute to the secretion of ECM proteins, leading to the development of fibrosis. The overall inhibition in expression of the *TGFB* and *SMAD-2/3* axis was aligned with decreased expression in downstream intracellular pathway genes, *ACTA2, CTGF*, and *COL* [46,47]. The reduced number of pSmad2-positive cells in FPF-treated skin samples, in comparison to untreated control, is a strong indication of reduced transcriptional modulation by TGF-β [48]. Furthermore, the lower number of αSMA-positive myofibroblast cells observed in the present study are suggestive of a decrease in myofibroblast differentiation. IFNγ, encoded by *IFNG*, is noted for its antagonistic effect on TGF-β, and its ability to inhibit collagen synthesis by myofibroblasts, characteristic of the fibrotic response to bleomycin injury [29]. A significant increase in *IFNG* expression in FPF-treated mice is in accord with lower TGF-β signaling and a diminished presence of αSMA-positive myofibroblast cells (Figure 4C–I). 

The valuable factors present in placental membranes, including a hyaluronic acid-rich matrix, play a major role in down-regulating TGF-β and its receptor expression by fibroblasts, and reducing myofibroblast differentiation [8,36]. A significant reduction in FSP1-positive fibroblasts was consistent with diminished ECM protein secretion (Figure 6B). FSP1, also known as S100A4, is considered a specific marker of fibroblasts undergoing tissue remodeling and is used as a biomarker to identify fibroblasts derived from EMT and monitor/predict mechanisms of tissue fibrosis [30]. As previous literature has provided evidence that all fibroblasts expressing S100A4 are likely to go undergo EMT, it is suggestive that the protein is involved in adult epithelial or endothelial cells’ transition to fibroblasts. Furthermore, as cell-lineage studies from various organs confirmed that S100A4-positive fibroblasts underwent EMT, S100A4 has now been accepted as an important hallmark of the EMT process [30,49]. A marked reduction in *S100A4* expression in FPF-treated mice is hence indicative of reduced EMT processes (Figure 6C). Constitutively downregulated *FLI1* is another influential target in dermal fibrosis and systemic sclerosis [31,32,50]. *FLI1* deficiency was shown to directly contribute to EndMT, and this process was shown to be further facilitated by bleomycin treatment [31,50]. Our observation of significantly increased expression of *FLI1* demonstrates a potential mechanism in reducing fibrosis via lowered EndMT (Figure 6D). Furthermore, inhibition of multifactorial *FLI1* is correlated with increased expression of *COL1A2*, *ACTA2*, *CTGF*, an increase in myofibroblast differentiation, altered expression of cell adhesion molecules, and M2 differentiation of macrophages [31,50,51]. Overall, the data is suggestive of a reduction in EMT and EndMT that is aggressively driven in chronic inflammatory and fibrotic tissue microenvironments. 

Directing therapeutics towards downregulating key inflammatory pathways has been a widely sought treatment for fibrosis. Bleomycin injury inevitably leads to an increased influx of inflammatory cells. Increased numbers of T cells and macrophages have been reported in scleroderma patients [52]. Since T cells actively participate in cell-cell contact with fibroblasts to promote fibrosis, we sought to assess the impact of FPF on T cell population upon bleomycin injury. The reduced number of CD3-positive T cells confirmed the positive effects of the anti-inflammatory cocktail-rich placental formulation [5,12]. In further support of these beneficial effects, macrophages, which are recruited via the production of pro-fibrotic cytokines such as TGF-β, were reduced in the FPF-treated dermis (Figure 5B). Macrophages are considered to be master regulators of fibrosis [1]. While macrophages facilitate tissue repair, M2 macrophages promote collagen deposition, eventually leading to fibrosis via TGF-β-mediated fibroblast recruitment and activation (Figure 5C). Moreover, activated M2 phenotype macrophages have been reported in the skin of scleroderma patients and implicated as a possible source of profibrotic cytokines [53]. Hence, single molecule therapies that modulate the recruitment and activation of distinct macrophages and T-cell populations have been studied [1]. Our findings demonstrate the potential of FPF to decrease the bleomycin-induced influx of inflammatory cells.

With the more recent advances in gene transcription and discovery of miRs, which have been shown to have a significant role in the regulation of gene expression, we decided to study the miR expression levels using Next-Generation Sequencing. MiRs are small evolutionarily-conserved noncoding RNAs involved in post-transcriptional regulation and have important roles in different pathophysiological processes. Dysregulation of several miRs identified as key players of inflammatory and cell regulatory pathways is believed to contribute to fibrotic disorders. Targets of many altered miRs have been validated and are suggested to be amenable to therapeutic modulations [54]. Recent literature has reported that the mechanism by which placental and umbilical tissue can positively affect target organs is via modulation of miR expression [5,11]. In our study, we observed that pro-inflammatory miR-347, responsible for regulating TNFα release, was down-regulated in mouse skin tissue treated with FPF [55]. IL-1R and TLRs are two major profibrotic and inflammatory mediators. Furthermore, both IL-1R-and TLR-initiated immune responses are strongly correlated with miR-147 expression [56,57]. In the present study, we observed a reduction in miR-147 expression in FPF-treated skin. These transcriptional changes support a decreased bleomycin-induced inflammatory tissue microenvironment. MiR-146 and miR-184 are two known critical transcriptional mediators of fibrosis. MiR-146 regulates fibrosis via the NFkB-mediated inflammatory signaling pathway and is a promising target for treating hepatic fibrosis [58]. Similarly, up-regulation of miR-184 promotes renal fibrosis by binding to HIF1AN [59]. We found that both miR-146 and miR-184 were down-regulated in the FPF treatment group. On the contrary, the up-regulation in miR-299 has been shown to protect against renal fibrosis via inhibition of TGF-β1-induced epithelial to mesenchymal transition [60]. Consistent with these data, we report an up-regulation in miR-299 and miR-376, previously known as miR-368. Previous studies have revealed the anti-proliferative and anti-migratory role of miR-376 in melanoma cells [61]. Moreover, both miR-376 and miR-299 inhibited cellular migration in PC3 and DU145 cells [62]. The up-regulation of miR-376 and miR-299 observed in the present study could possibly have an inhibitory effect on fibroblast migration and proliferation. Fetal tissue is an ideal model to study fibrosis and scarless tissue healing. In comparing fetal and adult lungs, both miR-299 and miR-376c are highly up-regulated in fetal lungs, suggesting that a reduction in miR-299 and mi-376c expression leads to aging-associated lung disease, such as idiopathic lung fibrosis [63]. Therefore, the observed increase in both miR-299 and miR-376 expression following FPF treatment likely contributes to the reduction in dermal fibrosis, and supports a potential therapeutic role for FPF. 

In summary, dermal fibrosis was induced in these studies by subdermal administration of multiple bleomycin injections in aged mice. The hypothesis that FPF can diminish the progression of fibrosis by modulating pro-fibrotic factors is supported by the present data. Furthermore, FPF is unique, as it constitutes the amnion, chorion, and umbilical tissue, hence providing combinatorial beneficial effects of endogenous viable cells, growth factors, and hyaluronic acid-rich ECM. Hence, the therapeutic effects of FPF, driven by multiple pathways, could potentially be extended to include multiple organs and a variety of disorders. 

## Supplementary Materials

Supplementary materials can be found at https://www.mdpi.com/1422-0067/21/12/4242/s1.
