# Supplementary material for: A Flowable Placental Formulation Prevents Bleomycin-Induced Dermal Fibrosis in Aged Mice
Source: Int J Mol Sci. 2020 Jun 14;21(12):4242. doi: 10.3390/ijms21124242 (PMC7352837; doi:10.3390/ijms21124242)

**Supplementary Table S1.** Twenty-five most significantly differentially-expressed microRNAs and annotation with log fold change (logFC) between groups “Control” and “FPF” p-values. N= 3 mice/group.


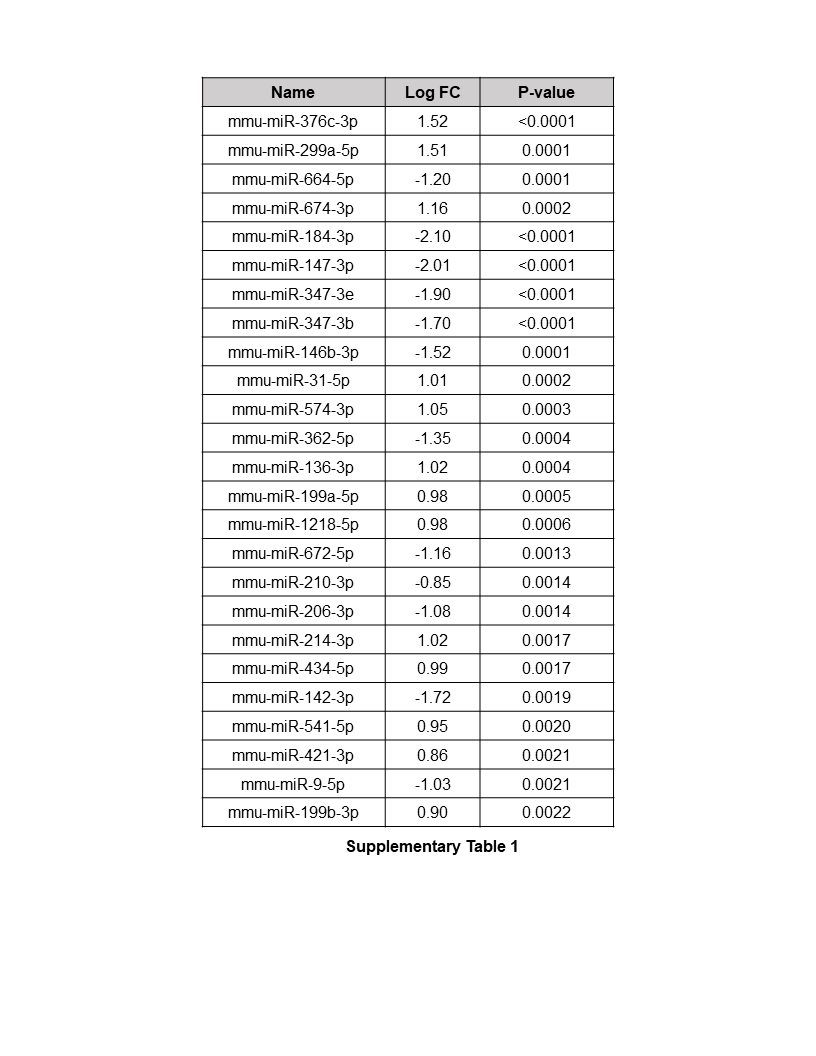

Supplement: Supplementary file 1 [file ijms-21-04242-s001.docx]
